# Differentially expressed genes, lncRNAs, and competing endogenous RNAs in Kawasaki disease

**DOI:** 10.7717/peerj.11169

**Published:** 2021-05-12

**Authors:** Changsheng Guo, Yuanqing Hua, Zuanhao Qian

**Affiliations:** 1 Department of Pediatrics, Affiliated Taikang Xianlin Drum Tower Hospital, Medical School of Nanjing University, Nanjing, China; 2 Nanjing Maigaoqiao Community Health Service Center, Nanjing, China

**Keywords:** Kawasaki disease, Microarray, Competing endogenous RNA, Long non-coding RNA

## Abstract

**Background:**

Kawasaki disease (KD) is an acute and febrile systemic vasculitis of unknown etiology. This study aimed to identify the competing endogenous RNA (ceRNA) networks of lncRNAs, miRNAs, and genes in KD and explore the molecular mechanisms underlying KD.

**Methods:**

GSE68004 and GSE73464 datasets were downloaded from the Gene Expression Omnibus. Differentially expressed lncRNAs (DElncRNAs) and genes (DEGs) in KD were identified using the criteria of *p* < 0.05 and | log_2_ (fold change) | ≥ 1. MicroRNAs (miRNAs) related to KD were searched from databases. The lncRNA-miRNA-mRNA networks involving the DElncRNAs and DEGs were constructed.

**Results:**

A total of 769 common upregulated, 406 common downregulated DEGs, and six DElncRNAs were identified in the KD samples. The lncRNA-miRNA-mRNA network consisted of four miRNAs, three lncRNAs (including the upregulated *PSORS1C3*, *LINC00999*, and the downregulated *SNHG5*) and four DEGs (including the downregulated *GATA3* and the upregulated *SOD2*, *MAPK14*, and *PPARG*). Validation in the GSE18606 dataset showed that intravenous immunoglobulin treatment significantly alleviated the deregulated profiles of the above RNAs in KD patients. Three ceRNA networks of *LINC00999-hsa-miR-6780-SOD2*, *PSORS1C3*-*hsa-miR-216a-PPARG/MAPK14*, and *SNHG5-hsa-miR-132*/*hsa*-*miR*-*92*-*GATA3* were identified. Four genes were associated with functional categories, such as inflammatory response and vascular endothelial cell.

**Conclusions:**

The ceRNA networks involve genes, such as *SOD2*, *MAPK14*, and *PPARG*, and lncRNAs, including* PSORS1C3*, *LINC00999*, and *SNHG5*, which might play a key role in the pathogenesis and development of KD by regulating inflammation.

## Introduction

Kawasaki disease (KD), also named mucocutaneous lymph node syndrome, is an acute, self-limiting, and febrile systemic vasculitis. The incidence of KD and rates of hospitalizations for KD are different across nations and ethnicities (Elakabawi et al., 2020; Holman et al., 2010; Kim et al., 2017; Lin & Wu, 2017). The incidence of KD is approximately 200 per 100,000 children <5 years old worldwide (Kim et al., 2017) and the KD-related hospitalization rate is approximately 20 per 100,000 children (Holman et al., 2010). KD predominantly affects children aged between 6 months and 5 years old. Intravenous immunoglobulin (IVIG) is the major treatment strategy for KD (Kim et al., 2017; Kim & Kim, 2016). The incidence of acute systemic vasculitis and acquired heart disease (including coronary artery abnormality) can be reduced by appropriate and timely treatment with IVIG and aspirin. However, the etiology of KD is largely unknown and the diagnosis of both complete and incomplete KD is challenging.

Many clinical and epidemiologic research studies suggest an infectious etiology for KD (Rowley, 2017; Shulman & Rowley, 2015). Viral respiratory infections are common in KD patients (Maggio, Fabiano & Corsello, 2019; Rosenfeld et al., 2020). KD is an immune-mediated echo of viral infection and viral infection might trigger KD (Rigante, 2020; Rosenfeld et al., 2020). The coronavirus disease (COVID-19) pandemic, characterized by profound hyperinflammation, leads to a missed or delayed diagnosis of KD (Jones et al., 2020; Ouldali et al., 2020; Roe, 2020; Toubiana et al., 2020). Jones et al. (2020) first presented a severe KD infant triggered by COVID-19 (SARS-CoV-2). SARS-CoV-2 infection contributes to a rapid increase in KD incidence (Ouldali et al., 2020). Also, many genetic factors, including genes, microRNAs (miRNA), and long non-coding RNAs (lncRNA), play crucial roles in KD and associate with IVIG resistance and coronary artery lesions (CAL) secondary to KD (Ahn et al., 2019; Kuo et al., 2017; Jones et al., 2020; Ko et al., 2019; Rong et al., 2017; Wang et al., 2019; Wright et al., 2018).

Genes, miRNAs, and lncRNAs play important roles in the regulation of many biological processes. LncRNAs affect gene regulation transcriptionally or post-transcriptionally by sponging miRNAs (Yan et al., 2015; Zhu et al., 2016). For instance, lncRNA myocardial infarction associated transcript (*MIAT*) regulates cardiac hypertrophy, angiogenesis, and endothelial cell function by sponging *miR-150* and *miR-150-5p* (Yan et al., 2015; Zhu et al., 2016). Also, the competing endogenous RNA (ceRNA) networks of lncRNAs trigger, control, or suppress disease conditions (Leisegang, 2018; Sun et al., 2019). However, there is insufficient information on the regulatory ceRNA networks of lncRNAs in KD. Additional evidence is required to probe into the clues of lncRNAs in KD.

This study was performed to investigate the ceRNA networks of differentially expressed lncRNAs (DElncRNAs) and differentially expressed genes (DEGs) in KD. DElncRNAs and DEGs in the blood samples from patients with KD were identified. The ceRNA mechanisms in KD were identified using integrated bioinformatics analysis of microarray datasets. This study might provide a reference for exploring the pathology of KD.

## Material and Methods

### Microarray data

The microarray datasets, GSE68004, GSE73464, and GSE18606, were downloaded from the Gene Expression Omnibus (GEO, http://www.ncbi.nlm.nih.gov/geo/) in August 2020. The GSE68004 dataset (GPL10558, Illumina HumanHT−12 V4.0 expression beadchip) contained 89 blood samples collected from 76 pediatric patients with complete KD, 13 pediatric patients with incomplete KD, and 37 blood samples from age- and sex-matched healthy controls. The GSE73464 dataset consisted of 839 samples, including 55 samples from healthy controls and 78 samples from patients with KD. The GSE18606 dataset was downloaded and 48 blood samples from nine healthy controls and 20 KD patients (eight IVIG non-responding and 12 IVIG-responding patients) at the acute and convalescent stages. The GSE68004 and GSE73464 datasets were used to screen DElncRNAs and DEGs and the GSE18606 dataset was used to validate the expression profiling.

### Data processing

The non-normalized raw data were downloaded and processed using the Limma package (Smyth, 2005). The expression levels of background-corrected and normalized probes were calculated. Probes mapped to human mRNAs and lncRNAs in the GRCh38 human reference genome were retained; otherwise, they were removed. In the case of multiple probes mapped to one mRNA or lncRNA, the mean expression value of the probes was calculated and regarded as the expression level of that mRNA or lncRNA.

### Analysis of differential expression

The DEGs and DElncRNAs in the KD samples were screened using the GEO2R analysis tool (http://www.ncbi.nlm.nih.gov/geo/geo2r/). Significant DEGs and DElncRNAs were identified using the criteria of *p* value <0.05 and —log_2_(fold change, FC)—≥1. DEGs and DElncRNAs with log_2_FC ≥1 were upregulated, and log_2_FC ≤ − 1 were downregulated, respectively. Common DEGs between the GSE68004 and GSE73464 datasets were retained and used for further analysis.

### Identification of KD-related genes databases

The Comparative Toxicogenomics Database (CTD, update 2019; http://ctdbase.org/) is a premier public resource consisting of literature-based and manually curated associations between diseases, genes, pathways, and chemicals (Davis et al., 2019). KD-related genes and Kyoto Encyclopedia of Genes and Genomes (KEGG) pathways were identified from the CTD database using the search keyword “mucocutaneous lymph node syndrome”. The genes and pathways that overlapped between DEGs and items in the CTD database were retained.

### Construction of the protein-protein interaction (PPI) network

The protein interaction pairs were identified in the STRING database (Version 11.0, https://string-db.org/cgi/input.pl) with a score >0.4. The PPI network was constructed using the Cytoscape software (version 3.8.0; https://cytoscape.org/) and network modules were identified using the Molecular Complex Detection (MCODE) plugin of Cytoscape.

### Functional enrichment analysis

The annotation of Gene Ontology biological processes and KEGG pathways presents the biological properties of DEGs. Gene Ontology biological processes and KEGG pathways related to DEGs were identified using the database for annotation, visualization, and integrated discovery (DAVID, version 6.7; https://david.ncifcrf.gov/) in this study. Significant enrichment was identified when the adjusted (BH correction) *p* value was <0.05.

### Identification of KD-related miRNAs

miRNAs related to DEGs in KD were searched in the miRWalk (http://mirwalk.umm.uni-heidelberg.de/), miRTarbase (http://mirtarbase.cuhk.edu.cn/php/index.php), and starBase (version 2.0 https://www.starbaserobins.org/our-programs/starbase-2-0/) databases. The miRNA-mRNA pairs identified from at least two databases were retained and used to construct the miRNA-mRNA regulatory network.

### Construction of the lncRNA-miRNA-mRNA ceRNA network

The miRNA-lncRNA pairs were obtained from the miRcode (http://www.mircode.org/), DIANA-LncBase v2 (http://carolina.imis.athena-innovation.gr/diana_tools/web/index.php?r=lncbasev2%2Findex-experimental), and starBase (version 2.0; https://www.starbaserobins.org/our-programs/starbase-2-0/) databases. LncRNA-miRNA pairs included in at least two databases were retained and used for the construction of the lncRNA-miRNA network. The ceRNA networks were subsequently constructed using the Cytoscape software.

### Functional clustering of the key items

GeneCLiP 3.0 (http://ci.smu.edu.cn/genclip3/analysis.php) is a web-based literature mining database providing the functional clustering of potential candidates. The hub DEGs and DElncRNAs in the ceRNA networks were subjected to GeneCLiP3.0. The heatmap of functional clustering was obtained with the criteria of *p* < 0.01 and hit ≥ 4.

## Results

### Identification of DEGs and DElncRNAs in KD

**Figure 1:**
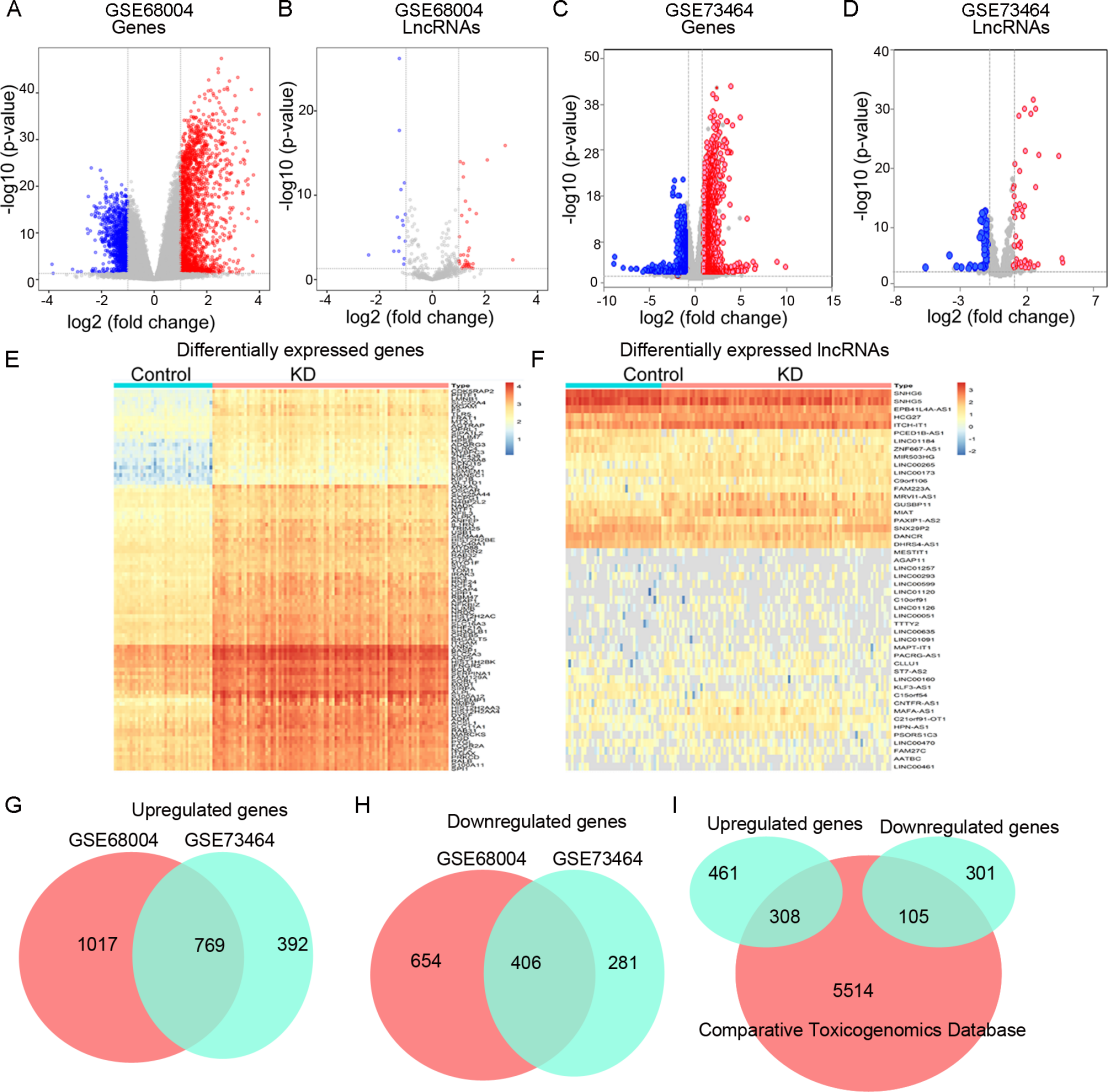
The differentially expressed genes (DEGs) and lncRNAs (DElncRNAs) in Kawasaki disease (KD). A and B, the Volcano plots of the DEGs and DElncRNAs between KD and control samples in the GSE68004 dataset, respectively. C and D, the Volcano plots of the DEGs and DElncRNAs in KD in the GSE73464 dataset, respectively. E and F, the heatmaps of the DEGs and DElncRNAs in the blood samples in the training GSE68004 dataset, respectively. G and H, the Venn diagrams indicating the common upregulated and downregulated DEGs between the two datasets, respectively. I, the Venn diagram identifying the shared genes between DEGs and the KD-related genes in the Comparative Toxicogenomics Database.

Based on the criteria of *p* value <0.05 and |logFC |≥1, a total of 2721 DEGs (1786 upregulated and 935 downregulated genes) and 1848 DEGs (1161 upregulated and 687 downregulated genes) were identified in the datasets GSE68004 and GSE73464, respectively (Figs. 1A and 1C). We also identified 48 DElncRNAs (36 upregulated and 12 downregulated) and 68 DElncRNAs (34 upregulated and 34 downregulated) in the GSE68004 and GSE73464 datasets, respectively (Figs. 1B and 1D). The DEGs and DElncRNAs showed distinctive expression profiles in the KD and control samples in the GSE68004 dataset (Figs. 1E and 1F).

Venn diagram identified 769 common upregulated and 406 common downregulated DEGs between the GSE68004 and GSE73464 datasets (Figs. 1G and 1H). In addition, 5927 KD-related genes were identified in the CTD, including 413 DEGs (308 upregulated and 105 downregulated genes; Fig. 1I; Table S1 ). Also, six common DElncRNAs were included in the two datasets, including four upregulated (*MRVI1*-*AS1*, *PSORS1C3*, *MAFA*-*AS1*, and *LINC00999*) and two downregulated lncRNAs in KD compared with controls (*SNHG5* and *KLF3*-*AS1*).

### Functional analysis of the DEGs

Functional enrichment analysis of the 413 common DEGs showed that the upregulated genes were significantly enriched with 279 biological processes including “GO:0006952∼ defense response”, “GO:0001817∼ regulation of cytokine production”, and “GO:0032675∼ regulation of interleukin-6 production”, and six KEGG pathways, including “hsa04060: Cytokine-cytokine receptor interaction”, “hsa04620: Toll-like receptor signaling pathway”, and “hsa04610: Complement and coagulation cascades” (Table S2). The downregulated genes were associated with 73 biological processes, including “GO:0042110∼ T cell activation”, “GO:0002694∼ regulation of leukocyte activation”, and “GO:0050870∼ positive regulation of T cell activation”, and six KEGG pathways, including “hsa05340: Primary immunodeficiency”, “hsa04660: T cell receptor signaling pathway”, and “hsa04060: Cytokine-cytokine receptor interaction” (Table S2).

**Figure 2:**
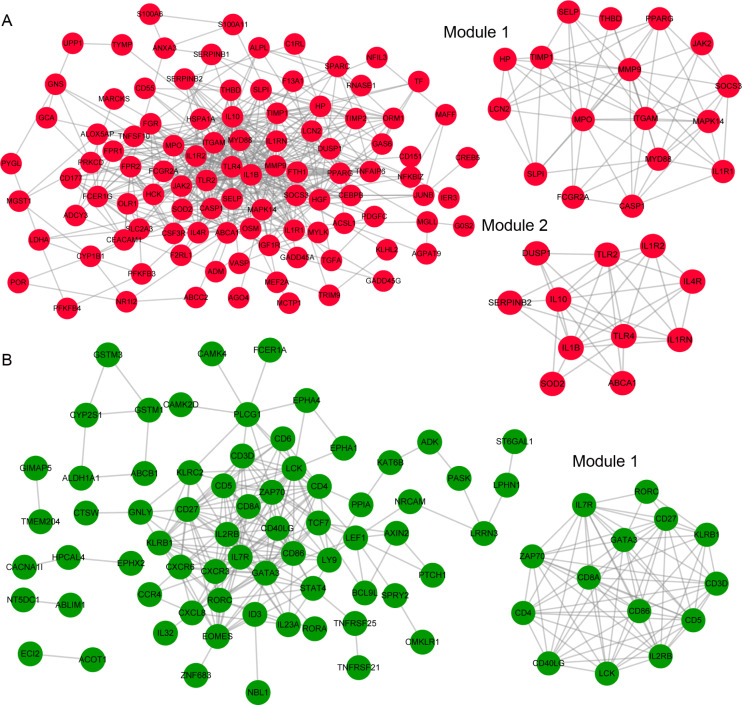
The modules and the protein–protein interaction (PPI) network of the differentially expressed genes (DEGs) in Kawasaki disease (KD). The protein-protein interaction (PPI) network and modules of the differentially expressed genes (DEGs) in Kawasaki disease (KD). (A & B) The PPI network and modules of the upregulated and downregulated DEGs, respectively. Red and green color notes the significant upregulation (*p* < 0.05 and log2Fold change ≥1) and downregulation (*p* < 0.05 and log2Fold change ≤ − 1), respectively.

### Construction of the PPI network and functional analysis

The PPI network of the upregulated genes consisted of 101 DEGs and 504 interaction pairs (Fig. 2A). We identified two modules (score >5) consisting of 17 and 11 upregulated genes in the upregulated PPI network (Table 1). The PPI network of the downregulated DEGs included 68 DEGs and 213 lines (Fig. 2B). One module consisting of 14 downregulated genes was included in the downregulated PPI network (Fig. 2B). The functional enrichment analysis showed that genes in module 1 of upregulated DEGs were enriched in 44 biological processes, including “GO:0009617∼ response to bacterium”, “GO:0032496∼ response to lipopolysaccharide”, “GO:0042981∼ regulation of apoptosis”, and “GO:0001817∼ regulation of cytokine production” (Table S3), and one KEGG pathway “hsa04670:Leukocyte transendothelial migration”. None functional categories enriched the DEGs in the other two modules. Genes in three PPI network modules were used to identify miRNA-target pairs.

**Table 1:** The list of the genes in the modules in the protein–protein network of the upregualted and downregulated genes in Kawasaki disease.

**Module**	**Genes**
Up-Module 1	MAPK14, THBD, PPARG, ITGAM, FCGR2A, HP, LCN2, SLPI, MPO, SELP, MYD88, IL1R1, MMP9, TIMP1, CASP1, JAK2, SOCS3
Up-Module 2	DUSP1, SERPINB2, SOD2, IL1B, IL1RN, IL4R, IL1R2, ABCA1, IL10, TLR4, TLR2
Down-Module 1	GATA3, IL2RB, CD27, CD40LG, CD3D, ZAP70, CD8A, KLRB1, IL7R, CD4, LCK, CD5, RORC, CD86

**Figure 3:**
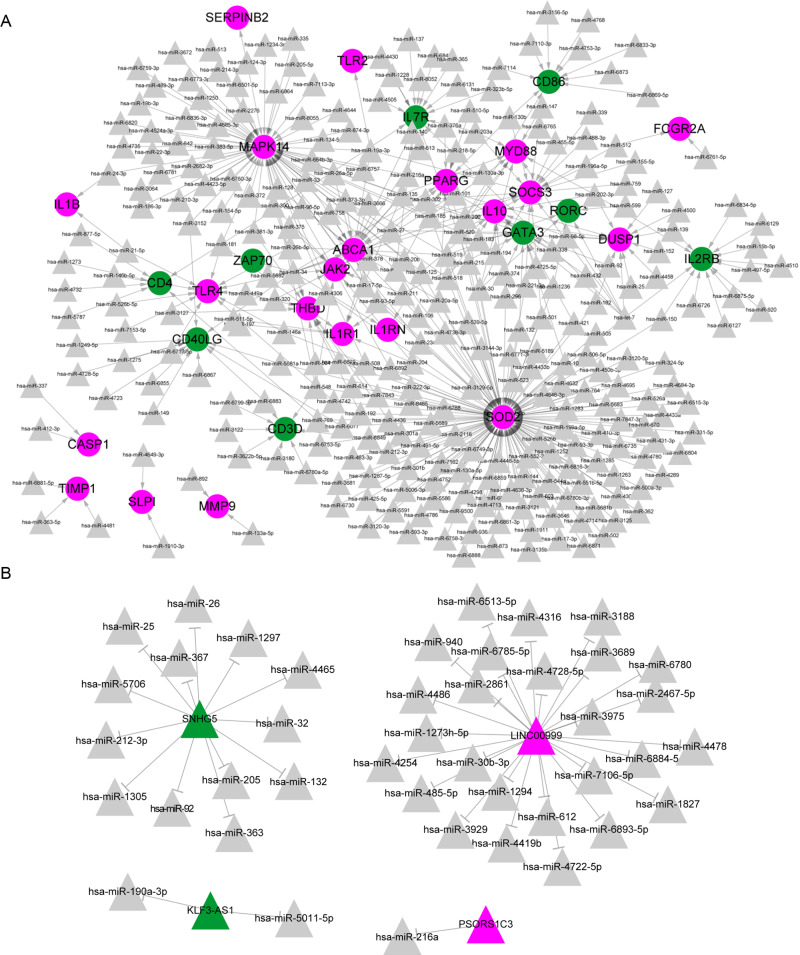
The predicted miRNA-mRNA and lncRNA-miRNA regulatory networks in Kawasaki disease (KD). The miRNA-mRNA and lncRNA-miRNA networks in Kawasaki disease (KD). (A) The miRNA-mRNA network consists of 12 differentially expressed genes (DEGs) and 257 miRNAs. (B) The lncRNA-miRNA network consists of 10 differentially expressed lncRNAs (DElncRNAs) and 79 miRNAs. The green and red colors note the downregulation and upregulation, respectively.

### Identification of ceRNAs networks

A total of 423 miRNA-target pairs were identified from databases, including 30 DEGs (nine downregulated and 21 upregulated genes) and 298 miRNAs in the miRNA-mRNA regulatory network (Fig. 3A). Also, we identified 42 lncRNA-miRNA pairs of four DElncRNAs from databases, including two upregulated lncRNAs (*PSORS1C3* and *LINC00999*) and two downregulated lncRNAs (*SNHG5* and *KLF3*-*AS1*).

**Figure 4:**
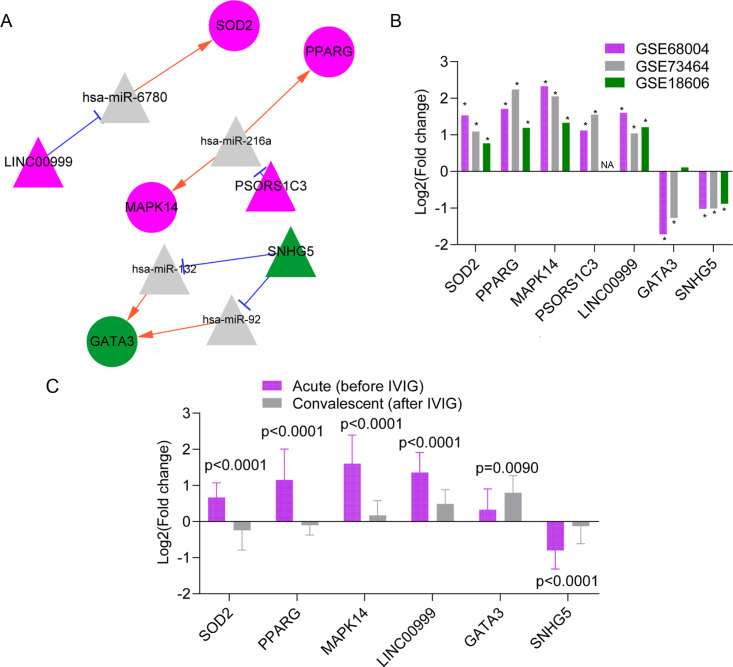
The predicted lncRNA-miRNA-mRNA regulatory networks in Kawasaki disease (KD). The lncRNA-miRNA-mRNA networks in Kawasaki disease (KD).(A) The lncRNA-miRNA-mRNA network contains four differentially expressed lncRNAs (DElncRNAs: two downregulated lncRNAs, green color; and two upregulated lncRNAs, red color), 11 miRNAs (gray color), and four differentially expressed genes (DEGs: three upregulated genes, red color; and one downregulated gene, green color). (B) The expression levels (log2Fold change values) of the seven RNAs in three datasets. Significant differences (p ¡ 0.05) are indicated by stars (*). NA: not detectable in the corresponding dataset. (C) The expression levels of RNAs in patients with KD before and after the intravenous immunoglobulin (IVIG) treatment. Data are expressed as mean ± standard deviation, and the differences are analyzed using the t-test.

According to the co-expression profiles of the DElncRNAs and DEGs, five lncRNA-miRNA-mRNA pairs were extracted from the lncRNA-miRNA and miRNA-mRNA pairs (Fig. 4A). The upregulated superoxide dismutase 2 (*SOD2*) gene was regulated by *LINC00999* through *hsa-miR-6780*. The upregulated genes peroxisome proliferator-activated receptor gamma (*PPARG*) and mitogen-activated protein kinase 14 (*MAPK14*) were regulated by lncRNA *PSORS1C3* through *hsa-miR-216a*. Besides, the downregulated lncRNA *SNHG5* regulated the GATA binding protein 3 (*GATA3*) gene through *hsa-miR-132* and *hsa-miR-92*.

### Microarray dataset validation of DEGs and DElncRNAs

Figure 4B presents the expression profiling of the seven DEGs and DElncRNAs in KD samples in microarray datasets. *LINC00999*, *SOD2*, *PPARG*, *PSORS1C3,* and *MAPK14* were upregulated in KD in all datasets, while *GATA3* and *SNHG5* were downregulated in KD samples in at least two datasets (Fig. 4B). We also observed that the IVIG treatment significantly attenuated the increased levels of *LINC00999*, *SOD2*, *PPARG*, and *MAPK14*, and increased the expression levels of *SNHG5* and *GATA3* in patients with KD (*p* < 0.05 by *t*-test, Fig. 4C). These results suggested that the lncRNA-miRNA-mRNA regulatory pairs, including the upregulated *LINC00999-hsa-miR-6780-SOD2* and *PSORS1C3*-*hsa-miR-216a-PPARG/MAPK14* networks and the downregulated *SNHG5-hsa-miR-132*/*hsa*-*miR*-*92*-*GATA3* network, might have crucial roles in the pathology of KD and treatment for KD.

### Functional clustering of the hub DElncRNAs and DEGs

The functional clustering of the hub DEGs and DElncRNAs in the lncRNA-miRNA-mRNA ceRNA network is shown in Fig. S1. *SNHG5* was associated with four items, including “acute myeloid leukemia”, “ovarian cancer”, “myeloid leukemia”, and “renal cell carcinoma”, and *PSORS1C3* was associated with two items, including “DNA methylation” and “psoriasis”. Four DEGs (*SOD2*, *GATA3*, *PPARG*, and *MAPK14*) were related to various functional categories, including “inflammatory response”, “cell activation”, “autoimmune disease”, and “vascular endothelial cell” (Fig. S1). These results indicated that DEGs and DElncRNAs were involved in various pathways.

## Discussion

The association of KD with COVID-19 provides a novel insight into the pathology of KD. Also, the associations of miRNAs and lncRNAs with pandemic COVID-19 suggested the key roles of them in COVID-19 management (Gambardella et al., 2020; Ramaiah, 2020; Teodori et al., 2020). Our study identified the significantly deregulated genes, lncRNAs, and ceRNA networks in KD. DEGs including *SOD2*, *GATA3*, *PPARG*, and *MAPK14* were associated with biological processes related to “inflammatory response”. LncRNAs including the downregulated *SNHG5* lncRNA and the upregulated *LINC00999* and *PSORS1C3* lncRNAs might have crucial roles in KD by regulating the above DEGs. Microarray validation showed that the IVIG treatment attenuated the expression of *SNHG5*, *LINC00999*, *SOD2*, *GATA3*, *PPARG*, and *MAPK14* in patients with KD, indicating the crucial roles of them in KD pathology and treatment.

Among the DElncRNAs in KD patients, *SNHG5* regulated *GATA3* by sponging *hsa-miR-132* and *hsa*-*miR*-*92*. *SNHG5* plays an important role in human tumors as an oncogenic lncRNA (Damas et al., 2016; Li et al., 2019b; Li et al., 2018; Zhang et al., 2019). *SNHG5* promotes tumor cell proliferation, survival, and drug resistance by sponging miRNAs to enhance gene expression (Damas et al., 2016; Li et al., 2019b; Li et al., 2018; Zhang et al., 2019). Zhang et al. (2019) showed that *SNHG5* is upregulated in colorectal cancer tissues and its expression increased cell proliferation, metastasis, and migration by inhibiting *miR-132-3p* and enhancing CAMP responsive element binding protein 5. Plasma *miR-132-5p* might be a diagnostic biomarker for early acute myocardial infarction (Li et al., 2019a). However, the inhibition of *miR-132* attenuates cortical inflammation (Mishra et al., 2017). Also, *miR-92* exhibits an anti-inflammatory effect and suppresses inflammatory responses in macrophages (Lai et al., 2013). Besides, the *GATA3* gene is an essential transcription factor and a critical regulator of immune cell function (Usary et al., 2004; Zhu et al., 2004). *GATA3* controls T helper type 2 (Th2) cell differentiation and Foxp3 ^+^ regulatory T cell fate (Wohlfert et al., 2011; Zhu et al., 2004). Th2 cells and the *GATA3* gene both were involved in airway inflammation (Choi et al., 2016; Jang et al., 2016; Peng et al., 2018). However, this is no direct information showing the association of *miR-132/92*, *SNHG5*, and *GATA3* with KD. Our study indicated that the expression levels of *SNHG5* and *GATA3* were downregulated in KD but were enhanced by the IVIG treatment. These results indicated that *SNHG5* and *GATA3* and the *SNHG5-hsa-miR-132*/*hsa*-*miR*-*92*-*GATA3* axis might have crucial roles in the pathology of KD through regulating inflammation.

Delayed diagnosis and treatment for KD may cause prolonged inflammation of vessel walls and a high risk for IVIG resistance and a high rate of CALs (Lech et al., 2019; Rigante, 2020; Türkuçar et al., 2020). Also, clinical variables, including the levels of platelet-derived microparticles, platelet count, and neutrophil count were associated with CALs (Chen et al., 2011; Hu et al., 2020; Jin et al., 2019). Molecular factors, including the Th2 cytokine thymus, activation-regulated chemokine/chemokine ligand 17 (*TARC/CCL1* 7) and the neutrophil hematopoietic cytokine granulocyte colony-stimulating factor (G-CSF) were related to IVIG resistance in KD (Abe et al., 2008; Lee et al., 2013). Patients with KD having an allele of the *TARC/CCL17* (rs4784805) had a better response to the IVIG treatment (Lee et al., 2013). Abe et al. (2008) showed that the serum G-CSF levels in IVIG nonresponsive patients were significantly higher than in responsive patients before treatment. These studies indicate the inflammatory biomarkers play critical roles in the pathogenesis of IVIG resistance and CALs in KD.

Oxidative stress contributes to inflammation and tissue injury. Elevated cardiac reactive oxygen species (ROS) accumulation is a common pathologic feature in KD and cardiac hypertrophy (Yahata & Hamaoka, 2017; Zhang et al., 2017). Neutrophil respiratory burst produces ROS and predicts the risk of CALs in KD (Hu et al., 2020). SOD2 is the primary antioxidant enzyme neutralizing •O2^−^ and its overexpression promotes reductive stress (Zhang et al., 2017). SOD2 prevents cardiac ROS production and hypertrophy features (Xie et al., 2020). These studies showed that *SOD2* upregulation might be a self-healing mechanism in KD. However, the associations of *hsa-miR-6780*, *SOD2*, and *LINC00999* with vasculitis and KD have not been reported till now. Also, microarray validation showed that IVIG treatment attenuated *SOD2* and *LINC00999* expression levels in the blood samples from patients with KD. These results showed that the ceRNA network of upregulated *SOD2* and *LINC00999* might protect against oxidative stress-induced damage in KD.

Another upregulated ceRNA network in KD was the *PSORS1C3*-*hsa-miR-216a-PPARG/MAPK14* network. *PPAR γ* is a nuclear hormone receptor predominantly expresses in adipose tissue and involves in adipogenesis (Fang et al., 2016; Son et al., 2007). Son et al. (2007) showed that *PPAR γ1* overexpression increased the expression of fatty acid oxidation genes in mouse hearts. Heart function could be improved by PPAR *γ* agonist (Fang et al., 2016; Vikramadithyan et al., 2005). PPAR *γ* is a target of anti-inflammatory drugs including the agonist thiazolidinediones which could ameliorate COVID-19 progression (Carboni, Carta & Carboni, 2020). Besides, MAPK14/p38 *α* regulates inflammatory response (Fazia et al., 2020; Wu et al., 2019). MAPK14 mediates autophagy and activates inflammation and proliferation in vascular smooth muscle cells (VSMCs) through the NF-kB signaling (Wu et al., 2019). Also, *miR-216a* has an anti-inflammatory effect in *in vitro* cell models (Kong et al., 2020; Tian et al., 2018; Yang et al., 2018). The upregulation of *miR-216a* or *miR-216a-5p* protects cells from oxidative stress-induced injury via targeting the NF- *κ*B and JAK signaling pathways (Kong et al., 2020; Tian et al., 2018; Yan et al., 2019; Yang et al., 2018). Microarray validation showed that the *PPARG* and *MAPK14* genes in KD were decreased following the IVIG treatment in the GSE18606 dataset. These results showed that the *PPARG* and *MAPK14* might be therapeutic targets for KD.

## Conclusions

In conclusion, we confirmed that the ceRNA networks, including the upregulated networks *LINC00999-hsa-miR-6780-SOD2* and *PSORS1C3*-*hsa-miR-216a-PPARG/MAPK14* and the downregulated network *SNHG5-hsa-miR-132*/*hsa*-*miR*-*92*-*GATA3*, might relate to the pathogenesis of and development of KD. These networks are associated with inflammation and response to IVIG treatment in KD. Our study provides new insights into the pathogenesis of KD. However, the ceRNA networks and their associations with KD should be validated.

##  Supplemental Information

10.7717/peerj.11169/supp-1 Figure S1 The heatmap of the functional clustering related to the genes and lncRNAs in the ceRNA network

10.7717/peerj.11169/supp-2 Table S1 The list of the shared terms between the differentially expressed genes and the Kawasaki disease-related genes in the Comparative Toxicogenomics Database

10.7717/peerj.11169/supp-3 Table S2 The list of the biological processes and pathways associated with the shared differentially expressed genes in the database and datasets

10.7717/peerj.11169/supp-4 Table S3 The list of the Gene Ontology (GO) biological processes and Kyoto Encyclopedia of Genes and Genomes (KEGG) pathways associated with the differentially expressed genes in the modules

## Additional Information and Declarations

### Data Availability

The following information was supplied regarding data availability: The original microarray data is available at the NCBI Gene Expression Omnibus: GSE68004, GSE18606 and GSE73464. All data generated during this study are contained in the figures and tables (Figs. 1– 4; Table 1; Tables S1– S3 and Fig. S1).
