# Supplementary figures and images for: Differentially expressed genes, lncRNAs, and competing endogenous RNAs in Kawasaki disease
Source: PeerJ. 2021 May 12;9:e11169. doi: 10.7717/peerj.11169 (PMC8123229; doi:10.7717/peerj.11169)

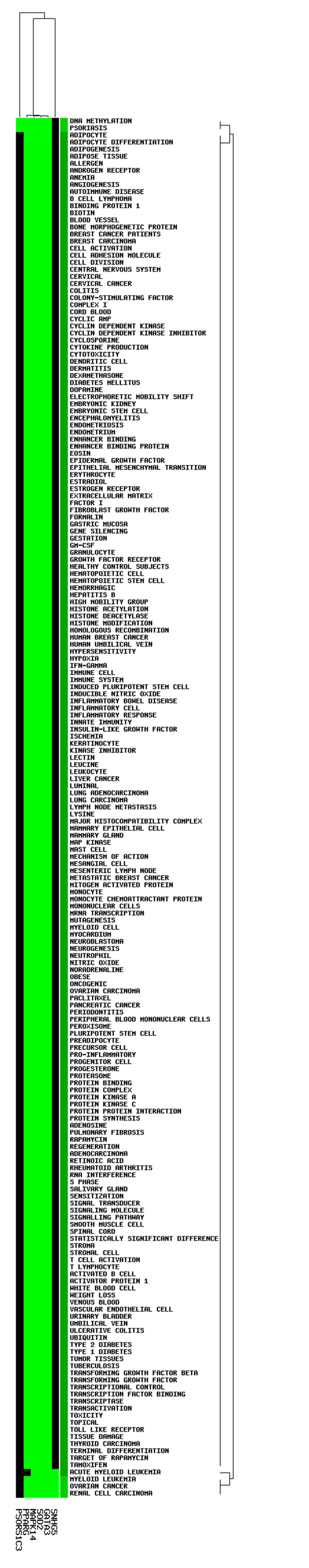

Supplement: Figure S1 [file peerj-09-11169-s001.jpg]
