# Supplementary material for: Differentially expressed genes, lncRNAs, and competing endogenous RNAs in Kawasaki disease
Source: PeerJ. 2021 May 12;9:e11169. doi: 10.7717/peerj.11169 (PMC8123229; doi:10.7717/peerj.11169)
Supplement: Table S1 [file peerj-09-11169-s002.docx]

Table S1. The list of the shared terms between the differentially expressed genes and the Kawasaki disease-related genes in the Comparative Toxicogenomics Database.

| Up | Up | Down |
| --- | --- | --- |
| FCGR2A | NUP214 | GSTM1 |
| IL1B | SBNO2 | BCL9L |
| TIMP2 | GALNT14 | SIGIRR |
| G0S2 | RAB24 | DGCR6 |
| TLR2 | VSIG10 | ST6GAL1 |
| IL1RN | ABCA13 | PTPRM |
| ABCA1 | CSTA | HKDC1 |
| HSPA1A | ITGA2B | CARNS1 |
| POR | PEAR1 | GSTM3 |
| TLR4 | CEP19 | NT5DC1 |
| PFKFB3 | CYB5R4 | SPRY2 |
| PPARG | NEDD1 | LMF1 |
| CD55 | COL7A1 | POLR3H |
| MARCKS | PROK2 | ECI2 |
| GADD45A | ST6GALNAC3 | CYP2S1 |
| TIMP1 | CYYR1 | LGALS2 |
| TUBA1A | ELANE | PPIA |
| ANXA3 | GNG10 | LARGE1 |
| TGFA | HOXA9 | MAL |
| MPO | ACOX1 | LEF1 |
| CSF3R | BRI3 | OCIAD2 |
| IL10 | ENTPD7 | IFFO2 |
| DUSP1 | GYG1 | GPM6B |
| IL4R | HDAC4 | ZAP70 |
| TNFSF10 | PDLIM7 | PLCG1 |
| PDGFC | INTU | CD4 |
| MMP9 | SLC2A14 | ADK |
| CREB5 | ALPK1 | CCR4 |
| SOD2 | SV2A | CACNA1I |
| ECE1 | TLR8 | CMKLR1 |
| TYMP | TREM1 | ZNF91 |
| VASP | FRAT1 | CTSW |
| GPAT3 | MANSC1 | CAMK2N1 |
| ABCC2 | RNF24 | ASB13 |
| SERPINB2 | RP2 | NBL1 |
| SELP | SGTB | SPOCK2 |
| IL1R1 | TSHZ3 | SH2D3A |
| IDI1 | FBXL5 | CD27 |
| MYD88 | LRRN1 | CXCR3 |
| JAK2 | NOD2 | LCK |
| ALPL | ANKRD22 | CXCL8 |
| PRKCD | CA4 | NDRG2 |
| LCN2 | MGAM | HOOK1 |
| JUNB | BBS9 | MAN1C1 |
| CD177 | SYTL4 | EPHX2 |
| MAPK14 | CDK5RAP2 | HPCAL4 |
| CEBPB | APMAP | EPHA4 |
| TRIM9 | LONRF3 | ADGRL1 |
| FPR2 | GNG5 | CD6 |
| SAMSN1 | PSTPIP2 | CD86 |
| C1RL | SUCNR1 | CD3D |
| KLHL2 | ATP9A | EPHA1 |
| TRPM6 | COL17A1 | TNFRSF25 |
| RNASE1 | DSE | PTCH1 |
| FPR1 | IMPDH1 | IL32 |
| TMEM144 | KCNJ15 | FBLN7 |
| AGO4 | RALGAPA2 | STAT4 |
| EIF4E3 | PGM2 | DBP |
| MYBPC3 | APH1B | CAPN5 |
| OSM | ETS2 | KLRC2 |
| GNS | NARF | TCF7 |
| CD151 | PIM3 | CD5 |
| ADCY3 | TLR5 | AXIN2 |
| GCA | TNNI2 | IL2RB |
| FGR | B3GNT5 | ABCB1 |
| MCTP1 | TSPAN2 | EBAG9 |
| SERPINB1 | MSRB3 | CAMK2D |
| ASPH | PILRA | RORC |
| MEF2A | ANPEP | SBK1 |
| DRAM1 | CPD | DUSP14 |
| FNDC3B | KCNE3 | ACOT1 |
| FCER1G | SMPDL3A | GIMAP5 |
| HCK | TCN2 | ID3 |
| F13A1 | BEX1 | ALDH1A1 |
| ALOX5AP | NCF2 | ITLN1 |
| PFKFB4 | SCN9A | EOMES |
| CYP1B1 | FLOT1 | TC2N |
| SLPI | LRPAP1 | CD8A |
| NR1I2 | PITX2 | TMEM204 |
| SULT1B1 | ACVRL1 | MMP28 |
| GAS6 | APBB2 | SYTL2 |
| CEACAM1 | C5AR1 | GATA3 |
| S100A6 | RHOBTB1 | TNFRSF21 |
| OLR1 | SERPINA1 | CAMK4 |
| F2RL1 | DUSP3 | NR3C2 |
| IL1R2 | EGF | PASK |
| PYGL | HRH2 | NRCAM |
| S100A11 | ITGAX | NCALD |
| NFKBIZ | PLPP2 | RORA |
| ORM1 | PTAFR | ABLIM1 |
| HP | SIRPA | PRSS23 |
| TNFAIP6 | ARHGAP26 | KAT6B |
| UPP1 | ITGB3 | CD40LG |
| HMGB2 | LRG1 | GNLY |
| MYLK | PARP9 | NELL2 |
| NFIL3 | SH3GLB1 | LY9 |
| SAT1 | CDC42EP3 | PDE9A |
| IER3 | ASAP1 | IL23A |
| THBD | CCPG1 | FEZ1 |
| MGLL | IGF2BP3 | KLRB1 |
| HGF | SEL1L | IL7R |
| SPARC | ARRB2 | PLA2G7 |
| GADD45G | ADAM9 | CXCR6 |
| SLC2A3 | GSN | LRRN3 |
| MGST1 | RAB20 | ZNF683 |
| ITGAM | SIPA1L2 | FCER1A |
| TF | GPCPD1 |  |
| ADM | BAZ1A |  |
| LDHA | ARRDC4 |  |
| ACSL1 | IL1RAP |  |
| FTH1 | NBN |  |
| MAFF | SAA1 |  |
| SOCS3 | TLR1 |  |
| IGF1R | CCR1 |  |
| CASP1 | RAP1GAP |  |
| GP6 | TP53I3 |  |
| LILRA2 | STX3 |  |
| FOLR3 | WSB1 |  |
| HSPA7 | RETN |  |
| NUMB | STOM |  |
| GKN1 | AQP9 |  |
| UBTD1 | BASP1 |  |
| GPR141 | NABP1 |  |
| TFF3 | PLOD2 |  |
| MS4A4A | RAB31 |  |
| NEDD4 | SLC1A3 |  |
| PPM1N | GRB10 |  |
| ANKRD35 | HHEX |  |
| LYPLA1 | TCF7L2 |  |
| RALB | BAMBI |  |
| CMTM5 | SMOX |  |
| OLFM4 | ADORA2B |  |
| F5 | BCL3 |  |
| BCL2A1 | CALD1 |  |
| KCNH3 | CAV2 |  |
| LILRA5 | ITGB5 |  |
| KIAA0930 | MXD1 |  |
| CLEC5A | GK |  |
| IFNGR2 | HPGD |  |
| FLOT2 | BCAT1 |  |
| TNFAIP8L3 | PGD |  |
| ABHD5 | BCL6 |  |
| ANGPTL3 | HSPA6 |  |
| ADGRG3 | NQO2 |  |
| C5AR2 | TRIB1 |  |
| FRAT2 | UBE2C |  |
| STAC | MYL9 |  |
| CMTM4 | TSPO |  |
| PICALM | ARG1 |  |
| CEACAM6 | SLC40A1 |  |
| SCN2B | VLDLR |  |
| KBTBD7 | NAMPT |  |
| FAR2 | ATF6 |  |
| NBEAL2 | TXN |  |
