# Supplementary material for: Differentially expressed genes, lncRNAs, and competing endogenous RNAs in Kawasaki disease
Source: PeerJ. 2021 May 12;9:e11169. doi: 10.7717/peerj.11169 (PMC8123229; doi:10.7717/peerj.11169)
Supplement: Table S2 [file peerj-09-11169-s003.docx]

Table S2. The list of the biological processes and pathways associated with the shared differentially expressed genes in the database and datasets.

| A, upregulated genes, Biological processes (Top 20) | | | | |
| --- | --- | --- | --- | --- |
| Term | Count | | PValue | Genes |
| GO:0009611~response to wounding | 41 | | 7.55E-14 | TF, ACVRL1, CCR1, F13A1, TLR1, F2RL1, TLR2, TLR4, ITGB3, TLR5, FPR2, IL10, TLR8, MYD88, GP6, SLC1A3, GSN, SAA1, IL1RAP, SCN9A, MGLL, IL1B, SERPINA1, NFKBIZ, SELP, CEBPB, OLR1, IL1RN, SOD2, HDAC4, ORM1, PROK2, TNFAIP6, CD55, THBD, F5, ADM, C1RL, SERPINB2, JAK2, PTAFR |
| GO:0006952~defense response | 43 | | 4.93E-13 | TF, IL1R1, ADORA2B, FGR, CCR1, TLR1, PPARG, TLR2, TLR4, HP, TLR5, FPR2, IL10, TLR8, NOD2, MYD88, LILRA2, SAA1, IL1RAP, SCN9A, CSF3R, MGLL, TFF3, IL1B, BCL3, SERPINA1, NFKBIZ, SELP, C5AR1, CEBPB, OLR1, NCF2, HCK, IL1RN, HDAC4, ORM1, TNFAIP6, PROK2, CD55, C1RL, MPO, CLEC5A, PTAFR |
| GO:0010033~response to organic substance | 44 | | 2.30E-11 | ALPL, ADCY3, CAV2, TF, HMGB2, IL1R1, CYP1B1, AQP9, PPARG, TLR2, TLR4, HSPA1A, IL10, TRIB1, IGF1R, NOD2, MYD88, ACSL1, GSN, HSPA6, IL1B, HSPA7, TFF3, SERPINA1, CASP1, GNG5, ADAM9, SELP, SOCS3, IL1RN, JUNB, ATF6, HDAC4, RETN, GRB10, THBD, DUSP1, ADM, MAPK14, GNG10, CA4, JAK2, MGST1, PTAFR, VLDLR |
| GO:0002237~response to molecule of bacterial origin | 16 | | 7.36E-11 | SELP, SOCS3, TLR2, TLR4, IL10, TRIB1, NOD2, THBD, MYD88, ADM, MAPK14, IL1B, SERPINA1, PTAFR, MGST1, VLDLR |
| GO:0006954~inflammatory response | 28 | | 1.38E-10 | TF, CCR1, TLR1, TLR2, TLR4, FPR2, TLR5, IL10, TLR8, MYD88, SAA1, IL1RAP, SCN9A, IL1B, MGLL, SERPINA1, SELP, NFKBIZ, CEBPB, OLR1, IL1RN, HDAC4, PROK2, TNFAIP6, ORM1, CD55, C1RL, PTAFR |
| GO:0032496~response to lipopolysaccharide | 15 | | 1.79E-10 | SELP, SOCS3, TLR4, IL10, TRIB1, NOD2, THBD, MYD88, ADM, MAPK14, IL1B, SERPINA1, PTAFR, MGST1, VLDLR |
| GO:0006955~immune response | 39 | | 3.64E-09 | IL1R2, NBN, IL1R1, SBNO2, AQP9, CCR1, TLR1, PPARG, TLR2, TLR4, TLR5, FTH1, IL10, TLR8, IGF1R, NOD2, MYD88, LILRA2, HRH2, IL4R, IL1RAP, LILRA5, FCER1G, IL1B, BCL3, NFIL3, C5AR1, CEBPB, OLR1, NCF2, IL1RN, PRKCD, OSM, CD55, TNFSF10, C1RL, TREM1, CLEC5A, PTAFR |
| GO:0001817~regulation of cytokine production | 19 | | 1.16E-08 | CEBPB, ADORA2B, PPARG, TLR1, ELANE, TLR2, TLR4, IGF2BP3, TLR8, IL10, NOD2, MYD88, SAA1, BCL3, IL1B, FCER1G, JAK2, BCL6, CASP1 |
| GO:0009617~response to bacterium | 19 | | 3.17E-08 | SELP, FGR, SOCS3, HCK, TLR2, TLR4, IL10, TRIB1, NOD2, MYD88, THBD, ADM, MAPK14, BCL3, IL1B, SERPINA1, PTAFR, MGST1, VLDLR |
| GO:0032680~regulation of tumor necrosis factor production | 8 | | 1.54E-06 | NOD2, MYD88, TLR1, TLR2, FCER1G, BCL3, TLR4, IL10 |
| GO:0050727~regulation of inflammatory response | 11 | | 1.75E-06 | OSM, SBNO2, ADORA2B, SAA1, PPARG, ELANE, FCER1G, BCL6, JAK2, TLR4, IL10 |
| GO:0002460~adaptive immune response based on somatic recombination of immune receptors built from immunoglobulin superfamily domains | 11 | | 1.98E-06 | CD55, NBN, NOD2, MYD88, C1RL, FCER1G, BCL3, TLR4, PRKCD, TLR8, IL10 |
| GO:0002250~adaptive immune response | 11 | | 1.98E-06 | CD55, NBN, NOD2, MYD88, C1RL, FCER1G, BCL3, TLR4, PRKCD, TLR8, IL10 |
| GO:0009725~response to hormone stimulus | 23 | | 2.27E-06 | ALPL, ADCY3, CAV2, HMGB2, SOCS3, PPARG, IL1RN, JUNB, IL10, RETN, IGF1R, GRB10, DUSP1, ADM, GNG10, CA4, IL1B, TFF3, JAK2, SERPINA1, GNG5, VLDLR, ADAM9 |
| GO:0007242~intracellular signaling cascade | 49 | | 2.42E-06 | ADCY3, HMGB2, NBN, ADORA2B, CCR1, TLR1, TLR2, FPR1, TLR4, TLR5, ABCA1, TLR8, IL10, TRIB1, IGF1R, NOD2, STAC, HRH2, GSN, RALB, RHOBTB1, TGFA, RAB24, BCL3, IL1B, EGF, GNG5, RAB20, ADAM9, C5AR1, SOCS3, HGF, UBE2C, PRKCD, MCTP1, OSM, WSB1, PROK2, RAB31, ADM, DUSP1, NEDD4, MAPK14, GNG10, GADD45G, JAK2, TREM1, APBB2, PTAFR |
| GO:0007243~protein kinase cascade | 23 | | 2.60E-06 | C5AR1, ADORA2B, SOCS3, TLR1, FPR1, TLR2, TLR4, TLR5, HGF, TLR8, IL10, TRIB1, OSM, PROK2, IGF1R, MAPK14, GADD45G, BCL3, IL1B, TGFA, JAK2, EGF, ADAM9 |
| GO:0009719~response to endogenous stimulus | 24 | | 3.31E-06 | ALPL, ADCY3, CAV2, HMGB2, SOCS3, PPARG, IL1RN, JUNB, IL10, RETN, IGF1R, GRB10, DUSP1, ADM, GNG10, CA4, TFF3, IL1B, JAK2, SERPINA1, GNG5, MGST1, VLDLR, ADAM9 |
| GO:0032675~regulation of interleukin-6 production | 8 | | 4.52E-06 | NOD2, CEBPB, ADORA2B, TLR1, TLR2, IL1B, TLR4, IL10 |
| GO:0016064~immunoglobulin mediated immune response | 9 | | 7.87E-06 | CD55, NBN, NOD2, MYD88, C1RL, FCER1G, BCL3, PRKCD, TLR8 |
| GO:0001819~positive regulation of cytokine production | 11 | | 8.29E-06 | NOD2, MYD88, ADORA2B, SAA1, TLR2, FCER1G, IL1B, BCL3, JAK2, TLR4, CASP1 |
| B, Upregulated genes, KEGG pathways | | | | |
| Term | | Count | PValue | Genes |
| hsa04640:Hematopoietic cell lineage | | 10 | 3.65E-04 | IL1R2, CD55, IL1R1, IL4R, IL1B, CSF3R, ANPEP, ITGB3, ITGAM, ITGA2B |
| hsa04060:Cytokine-cytokine receptor interaction | | 15 | 8.08E-03 | IL1R2, IL1R1, ACVRL1, CCR1, HGF, IL10, OSM, TNFSF10, IL4R, IL1RAP, CSF3R, IL1B, PDGFC, EGF, IFNGR2 |
| hsa04620:Toll-like receptor signaling pathway | | 8 | 1.60E-02 | MYD88, MAPK14, TLR1, TLR2, IL1B, TLR4, TLR5, TLR8 |
| hsa04610:Complement and coagulation cascades | | 6 | 3.36E-02 | CD55, C5AR1, THBD, F5, F13A1, SERPINA1 |
| hsa04510:Focal adhesion | | 11 | 3.68E-02 | IGF1R, CAV2, ITGB5, PDGFC, HGF, ITGB3, EGF, VASP, MYLK, ITGA2B, MYL9 |
| hsa04666:Fc gamma R-mediated phagocytosis | | 7 | 3.73E-02 | GSN, HCK, ASAP1, MARCKS, FCGR2A, PRKCD, VASP |
| C, Downregulated genes, biological processes (Top 20) | | | | |
| Term | Count | | PValue | Genes |
| GO:0042110~T cell activation | 10 | | 1.10E-07 | CD86, IL23A, GIMAP5, CD3D, CD8A, LCK, ZAP70, EOMES, CD4, IL7R |
| GO:0030217~T cell differentiation | 8 | | 1.83E-07 | GIMAP5, CD3D, CD8A, LCK, ZAP70, EOMES, CD4, IL7R |
| GO:0030098~lymphocyte differentiation | 9 | | 3.05E-07 | GIMAP5, CD3D, CD8A, CD40LG, LCK, ZAP70, EOMES, CD4, IL7R |
| GO:0002696~positive regulation of leukocyte activation | 9 | | 3.81E-07 | FCER1A, CD86, GIMAP5, LCK, ZAP70, CD4, IL7R, CD5, CD27 |
| GO:0050867~positive regulation of cell activation | 9 | | 5.44E-07 | FCER1A, CD86, GIMAP5, LCK, ZAP70, CD4, IL7R, CD5, CD27 |
| GO:0046649~lymphocyte activation | 11 | | 5.66E-07 | CD86, IL23A, GIMAP5, CD3D, CD8A, CD40LG, LCK, ZAP70, EOMES, CD4, IL7R |
| GO:0002694~regulation of leukocyte activation | 10 | | 1.15E-06 | FCER1A, CD86, GIMAP5, LCK, ZAP70, CD4, RORA, IL7R, CD5, CD27 |
| GO:0050865~regulation of cell activation | 10 | | 1.78E-06 | FCER1A, CD86, GIMAP5, LCK, ZAP70, CD4, RORA, IL7R, CD5, CD27 |
| GO:0002521~leukocyte differentiation | 9 | | 1.92E-06 | GIMAP5, CD3D, CD8A, CD40LG, LCK, ZAP70, EOMES, CD4, IL7R |
| GO:0051251~positive regulation of lymphocyte activation | 8 | | 2.87E-06 | CD86, GIMAP5, LCK, ZAP70, CD4, IL7R, CD5, CD27 |
| GO:0045321~leukocyte activation | 11 | | 3.34E-06 | CD86, IL23A, GIMAP5, CD3D, CD8A, CD40LG, LCK, ZAP70, EOMES, CD4, IL7R |
| GO:0006955~immune response | 17 | | 7.03E-06 | ST6GAL1, TCF7, CD8A, CMKLR1, EOMES, IL32, LY9, IL7R, SIGIRR, CTSW, CD86, IL23A, CD40LG, CCR4, ZAP70, CD4, CD27 |
| GO:0050870~positive regulation of T cell activation | 7 | | 9.06E-06 | CD86, GIMAP5, LCK, ZAP70, CD4, IL7R, CD5 |
| GO:0001775~cell activation | 11 | | 1.50E-05 | CD86, IL23A, GIMAP5, CD3D, CD8A, CD40LG, LCK, ZAP70, EOMES, CD4, IL7R |
| GO:0051249~regulation of lymphocyte activation | 8 | | 4.58E-05 | CD86, GIMAP5, LCK, ZAP70, CD4, IL7R, CD5, CD27 |
| GO:0045621~positive regulation of lymphocyte differentiation | 5 | | 7.18E-05 | CD86, GIMAP5, ZAP70, IL7R, CD27 |
| GO:0050863~regulation of T cell activation | 7 | | 1.05E-04 | CD86, GIMAP5, LCK, ZAP70, CD4, IL7R, CD5 |
| GO:0030097~hemopoiesis | 9 | | 1.36E-04 | GIMAP5, CD3D, CD8A, CD40LG, LCK, ZAP70, EOMES, CD4, IL7R |
| GO:0002684~positive regulation of immune system process | 9 | | 1.44E-04 | FCER1A, CD86, GIMAP5, LCK, ZAP70, CD4, IL7R, CD5, CD27 |
| GO:0050850~positive regulation of calcium-mediated signaling | 4 | | 1.64E-04 | FCER1A, CD8A, ZAP70, CD4 |
| D, Downregulated gene, KEGG pathways | | | | |
| Term | | Count | PValue | Genes |
| hsa05340:Primary immunodeficiency | | 7 | 9.79E-07 | CD3D, CD8A, CD40LG, LCK, ZAP70, CD4, IL7R |
| hsa04660:T cell receptor signaling pathway | | 7 | 6.61E-04 | PLCG1, CD3D, CD8A, CD40LG, LCK, ZAP70, CD4 |
| hsa04060:Cytokine-cytokine receptor interaction | | 10 | 1.01E-03 | TNFRSF21, IL2RB, IL23A, CD40LG, TNFRSF25, CCR4, CXCR6, CXCR3, IL7R, CD27 |
| hsa04514:Cell adhesion molecules (CAMs) | | 7 | 1.88E-03 | NRCAM, CD86, PTPRM, CD8A, CD40LG, CD4, CD6 |
| hsa04640:Hematopoietic cell lineage | | 5 | 1.04E-02 | CD3D, CD8A, CD4, IL7R, CD5 |
| hsa05217:Basal cell carcinoma | | 4 | 1.73E-02 | TCF7, LEF1, PTCH1, AXIN2 |
