# Supplementary material for: Differentially expressed genes, lncRNAs, and competing endogenous RNAs in Kawasaki disease
Source: PeerJ. 2021 May 12;9:e11169. doi: 10.7717/peerj.11169 (PMC8123229; doi:10.7717/peerj.11169)
Supplement: Table S3 [file peerj-09-11169-s004.docx]

Table S3. The list of the Gene Ontology (GO) biological processes and Kyoto Encyclopedia of Genes and Genomes (KEGG) pathways associated with the differentially expressed genes in the upregulated module 1.

| A, Biological processes |  |  |  |
| --- | --- | --- | --- |
| Term | Count | PValue | Genes |
| GO:0010033~response to organic substance | 9 | 1.41E-07 | SELP, IL1R1, MYD88, THBD, SOCS3, MAPK14, PPARG, JAK2, CASP1 |
| GO:0032496~response to lipopolysaccharide | 5 | 9.30E-07 | SELP, MYD88, THBD, SOCS3, MAPK14 |
| GO:0002237~response to molecule of bacterial origin | 5 | 1.45E-06 | SELP, MYD88, THBD, SOCS3, MAPK14 |
| GO:0009617~response to bacterium | 5 | 3.59E-05 | SELP, MYD88, THBD, SOCS3, MAPK14 |
| GO:0030099~myeloid cell differentiation | 4 | 1.08E-04 | MMP9, PPARG, JAK2, TIMP1 |
| GO:0006952~defense response | 6 | 2.72E-04 | SELP, IL1R1, MYD88, PPARG, MPO, HP |
| GO:0001817~regulation of cytokine production | 4 | 7.69E-04 | MYD88, PPARG, JAK2, CASP1 |
| GO:0042981~regulation of apoptosis | 6 | 9.34E-04 | MYD88, SOCS3, MMP9, MPO, JAK2, CASP1 |
| GO:0043067~regulation of programmed cell death | 6 | 9.77E-04 | MYD88, SOCS3, MMP9, MPO, JAK2, CASP1 |
| GO:0010941~regulation of cell death | 6 | 9.93E-04 | MYD88, SOCS3, MMP9, MPO, JAK2, CASP1 |
| GO:0045597~positive regulation of cell differentiation | 4 | 0.001518 | SOCS3, MAPK14, PPARG, JAK2 |
| GO:0030097~hemopoiesis | 4 | 0.001655 | MMP9, PPARG, JAK2, TIMP1 |
| GO:0048534~hemopoietic or lymphoid organ development | 4 | 0.002183 | MMP9, PPARG, JAK2, TIMP1 |
| GO:0031099~regeneration | 3 | 0.002243 | SOCS3, PPARG, JAK2 |
| GO:0002520~immune system development | 4 | 0.002588 | MMP9, PPARG, JAK2, TIMP1 |
| GO:0051094~positive regulation of developmental process | 4 | 0.002642 | SOCS3, MAPK14, PPARG, JAK2 |
| GO:0034097~response to cytokine stimulus | 3 | 0.002928 | IL1R1, MYD88, SOCS3 |
| GO:0001819~positive regulation of cytokine production | 3 | 0.003781 | MYD88, JAK2, CASP1 |
| GO:0045087~innate immune response | 3 | 0.008676 | IL1R1, MYD88, PPARG |
| GO:0045123~cellular extravasation | 2 | 0.010304 | SELP, ITGAM |
| GO:0043434~response to peptide hormone stimulus | 3 | 0.010711 | SOCS3, PPARG, JAK2 |
| GO:0032101~regulation of response to external stimulus | 3 | 0.011386 | SELP, PPARG, JAK2 |
| GO:0006928~cell motion | 4 | 0.011729 | SELP, MAPK14, JAK2, ITGAM |
| GO:0010740~positive regulation of protein kinase cascade | 3 | 0.012506 | MYD88, JAK2, CASP1 |
| GO:0030334~regulation of cell migration | 3 | 0.012793 | SELP, MMP9, JAK2 |
| GO:0032731~positive regulation of interleukin-1 beta production | 2 | 0.014398 | JAK2, CASP1 |
| GO:0009611~response to wounding | 4 | 0.015762 | SELP, MYD88, THBD, JAK2 |
| GO:0040012~regulation of locomotion | 3 | 0.016301 | SELP, MMP9, JAK2 |
| GO:0051270~regulation of cell motion | 3 | 0.016462 | SELP, MMP9, JAK2 |
| GO:0032732~positive regulation of interleukin-1 production | 2 | 0.017458 | JAK2, CASP1 |
| GO:0070555~response to interleukin-1 | 2 | 0.017458 | IL1R1, MYD88 |
| GO:0006916~anti-apoptosis | 3 | 0.018617 | MYD88, SOCS3, MPO |
| GO:0032651~regulation of interleukin-1 beta production | 2 | 0.02051 | JAK2, CASP1 |
| GO:0032652~regulation of interleukin-1 production | 2 | 0.023552 | JAK2, CASP1 |
| GO:0051240~positive regulation of multicellular organismal process | 3 | 0.025561 | MYD88, JAK2, CASP1 |
| GO:0010627~regulation of protein kinase cascade | 3 | 0.026543 | MYD88, JAK2, CASP1 |
| GO:0031100~organ regeneration | 2 | 0.026586 | SOCS3, PPARG |
| GO:0007159~leukocyte adhesion | 2 | 0.028604 | SELP, ITGAM |
| GO:0002573~myeloid leukocyte differentiation | 2 | 0.034634 | MMP9, PPARG |
| GO:0009967~positive regulation of signal transduction | 3 | 0.036284 | MYD88, JAK2, CASP1 |
| GO:0042592~homeostatic process | 4 | 0.039202 | PPARG, HP, JAK2, TIMP1 |
| GO:0007259~JAK-STAT cascade | 2 | 0.039632 | SOCS3, JAK2 |
| GO:0042127~regulation of cell proliferation | 4 | 0.044131 | MYD88, PPARG, JAK2, TIMP1 |
| GO:0010647~positive regulation of cell communication | 3 | 0.044253 | MYD88, JAK2, CASP1 |
| B, KEGG pathways |  |  |  |
| Term | Count | PValue | Genes |
| hsa04670:Leukocyte transendothelial migration | 3 | 0.025612 | MAPK14, MMP9, ITGAM |
